# Incidence of Hirschsprung Disease at the Central Pediatrics Teaching Hospital in Iraq: A Pathological Overview

**DOI:** 10.2174/0118715303401696251002054047

**Published:** 2025-10-20

**Authors:** Ikram Fakhri Abed Al-Zughaibi, Nada K. Mehdi, Hany Akeel Al-hussaniy

**Affiliations:** 1 Pathologist/Arab Board of Health Specializations in Anatomic Pathology (ABHS-APath), Al Zahraa Teaching Hospital, Kut, Iraq;; 2 Department of Pharmacology, College of Pharmacy, University of Al-Nisour, Baghdad, 10001, Iraq;; 3 Senior Pathologist/Central Pediatrics Teaching Hospital, Baghdad, Iraq;; 4 Iraqi Medical Research Center, Baghdad, Iraq

**Keywords:** Hirschsprung disease, mutation, missense, congenital disorder, Iraqi pediatric cohort, rectal biopsy

## Abstract

**Background:**

Hirschsprung disease (HD) is a congenital disorder associated with specific missense mutations in the RET proto-oncogene. This study aimed to demonstrate the incidence of Hirschsprung disease and its clinical and pathological aspects in an Iraqi pediatric cohort from a major referral hospital in Baghdad.

**Methods:**

A retrospective analysis was conducted over a ten-year period, reviewing the clinical and surgical records of patients diagnosed with Hirschsprung disease. Pathological sections were re-evaluated, and patient medical histories, prior surgeries, and other relevant clinical data were confirmed.

**Results:**

A total of 106 cases of Hirschsprung disease were identified. The mean age at diagnosis was 2.4 ± 3.0 years, with 40.6% of cases diagnosed within the first year of life. The male-to-female ratio was 2.6:1. The most commonly affected anatomical sites were the colon (35.8%) and rectum (23.6%). Pathological evaluation revealed the absence of ganglion cells in 57.5% of cases. Rectal biopsy was the most frequently performed diagnostic procedure (64.2%), and colon resection was required in 35.8% of cases. A significant association was found between disease presence and anatomical site involvement (*P* = 0.010) and surgical intervention (*P* = 0.046).

**Conclusion:**

The study highlights a male predominance in Hirschsprung disease, with the majority of cases diagnosed within the first year of life. The rectum and colon were the most commonly affected sites. Significant associations were observed between disease presence and anatomical site involvement, as well as surgical interventions, emphasizing the importance of early diagnosis and appropriate management strategies.

## 1 INTRODUCTION

Hirschsprung disease (HD) is characterized by congenital absence of ganglion cells and neuronal elements in variable segments of the intestinal tract, resulting in motility disturbances of the bowel [1-4]. It has been reported worldwide with relatively consistent incidence rates, but occurs less frequently in association with familial, parental, or sibling history, or with the concurrent development of other congenital anomalies [5-7]. HD represents a significant cause of colorectal obstruction, particularly in neonates and infants, and although it has already been delineated, multiple controversies exist concerning embryogenesis, manifestations, etiology, and pathogenesis [8-10]. Hirschsprung’s disease (HD) is caused by the arrest of migration of neuroblasts in the large bowel, leading to an absence of the neuroblasts in the myenteric plexus and a subsequent peristalsis of the affected bowel segment. Genetic and environmental factors have been previously incriminated in the pathogenesis of HD [11-14].

The absence or reduction in size and organization of the submucosal ganglion of Meissner and the myenteric plexus of Auerbach is considered a well-established feature of this entity [15]. The new generation of acronyms developed for this disease reflects the recent achievements and technical advances made in its understanding, including immunohistochemical studies [16-20]. Recently, a new tool, the cup biopsy, enables objective screening based on the identification by routine stains and subsequent verification of the submucosal plexus network, thereby minimizing the number of false diagnoses in short histological specimens. The problem has also been addressed from a genetic perspective, and a gene for the short segment has been named at the level of chromosome 10 [21-23].

Globally, the incidence of Hirschsprung disease is approximately 1 in 5,000 live births. Between 75% and 80% of individuals with Hirschsprung disease have a short segment at the end of the aganglionic part of the colon, located near the anus. Mutations in the RET (Rearranged during Transfection) proto-oncogene, the gene most commonly implicated in HD, exhibit higher penetrance in males than in females. This means that males who carry an RET mutation are more likely to develop the disease compared to females with the same mutation. The reduced penetrance in females may explain their lower incidence, despite carrying pathogenic variants [24-26].

### 1.1 Hirschsprung Disease: Pathophysiology and Clinical Presentation

More recently, a major advance was made in the genetics of HD, and the genetic alterations were scrutinized; however, the precise pathogenesis of the disease remains unresolved. Even though many have designated the etiology of HD to hypoplasia of enteric ganglia, the degree and prevalence of enteric ganglion cell injury, as well as the presence of hypertrophic nerve fibers, were evident. It is still uncertain if hypoplasia or neural damage is crucial for aganglionosis [27-32].

### 1.2 Histopathological Examination

Histopathological examination is necessary for diagnosing Hirschsprung disease (HD) and excluding other conditions, such as chronic aganglionosis and isolated hypoganglionosis, as they require different management and have different outcomes [33]. On hematoxylin and eosin staining, ganglion cells appear as large, spindle, or pyramidal cells with eosinophilic cytoplasm and oval or elongated nuclei, and they are normally distributed throughout the myenteric plexus or in the submucosal or myenteric plexus-nerve bundles [34-36].

### 1.3 Genetic and Molecular Pathogenesis of Hirschsprung Disease

Hirschsprung Disease (HD) is a complex congenital disorder resulting from the failure of neural crest cells to migrate, proliferate, or differentiate appropriately during the embryological development of the enteric nervous system [37-39]. Among the most well-established genetic contributors to HD are mutations in the RET proto-oncogene and the endothelin receptor type B gene (EDNRB), both of which play pivotal roles in enteric neurogenesis [40].

The RET gene, located on chromosome 10q11.2, encodes a receptor tyrosine kinase that is critical for the survival, proliferation, and migration of neural crest-derived cells. Mutations in RET are the most common genetic cause of HD, found in approximately 50% of familial cases and 15–20% of sporadic cases, particularly in short-segment disease. RET mutations can lead to reduced or absent signaling required for the proper colonization of the bowel by enteric neurons [41].

## 2 MATERIALS AND METHODS

### 2.1 Study Design and Setting

This retrospective study was conducted at the Central Pediatrics Teaching Hospital in Baghdad, Iraq, over a 10-year period. The study aimed to assess the incidence and pathological features of Hirschsprung disease among pediatric patients.

### 2.2 Study Population

The study included all pediatric patients diagnosed with Hirschsprung disease during the study period. The study spanned 10 years and included pediatric patients aged from 1 day to 14 years, of both sexes, who were diagnosed with Hirschsprung disease. A total of 106 eligible participants were included, all of whom met the diagnostic inclusion criteria (Table **S1**).

### 2.3 Inclusion and Exclusion Criteria

- Inclusion criteria: Pediatric patients with a confirmed diagnosis of Hirschsprung disease based on clinical, surgical, and histopathological findings.
- Exclusion criteria: Patients with incomplete records or coexisting congenital anomalies that may affect diagnostic interpretation.

### 2.4 Data Collection

Data were collected through a comprehensive review of hospital records, including patient demographics, clinical presentation, surgical interventions, and pathological reports. The collected information included:

- Age at diagnosis
- Gender distribution
- Clinical symptoms and signs
- Types of surgical procedures performed
- Histopathological findings

### 2.5 Pathological Examination

Histopathological evaluation was conducted on biopsy samples obtained from patients with the disease. The specimens were obtained from the mucosa-free submucosa layer of the gut. The tissue specimens were fixed for 24 to 48 hours in buffered formalin and then embedded in paraffin. Paraffin-embedded specimens were cut at 5 µm, mounted on glass slides, and stained with hematoxylin and eosin. These preoperative confirmatory results accurately predict the presence of the aganglionic colon. However, the short-segment disease may cause a false-negative finding on rectal biopsy [42, 43]. Hematoxylin and eosin (H&E) staining was used to assess the presence or absence of ganglion cells, with additional immunohistochemical staining performed when required. The pathological review included re-evaluation of archived slides to confirm the diagnosis [44].

### 2.6 Sample Size

The sample size (n = 106) was based on the total number of eligible patients diagnosed with Hirschsprung disease at our institution over 10 years [45]. As this is a retrospective study utilizing available medical records, formal sample size estimation using prevalence-based formulas was not applicable. Instead, we included all confirmed cases during the study period to maximize statistical power and representativeness [46].

### 2.7 Randomization, Blinding, and Controls

As a retrospective observational study, randomization and blinding were not applicable, and no control group was included. All patients meeting the inclusion criteria were analyzed descriptively and comparatively.

### 2.8 SAGER Guidelines Compliance

The authors followed the Sex and Gender Equity in Research (SAGER) Guidelines.

### 2.9 Statistical Analysis

The collected data were analyzed using IBM SPSS-29. Descriptive statistics were presented as frequencies, percentages, means, standard deviations, and ranges. Comparative analyses were conducted using the Pearson Chi-square test for categorical variables and the Student's t-test for continuous variables. Statistical significance was considered at *P*≤0.05.

## 3 RESULTS

### 3.1 Demographic Characteristics

A total of 106 patients diagnosed with Hirschsprung disease were included in this study. The mean age of the patients was 2.4±3.0 years (range: 1 day to 14 years). The majority of cases (40.6%) were diagnosed within the first year of life, followed by 30.2% between 1 and 3 years, and 17.9% between 3 and 6 years. Only a small proportion (6.6%) were diagnosed at 9 years or older. Regarding gender distribution, males comprised 72.6% (n=77) of the cases, while females accounted for 27.4% (n=29), resulting in a male-to-female ratio of approximately 2.6:1.

### 3.2 Age Distribution by Months

**Fig. (1):**
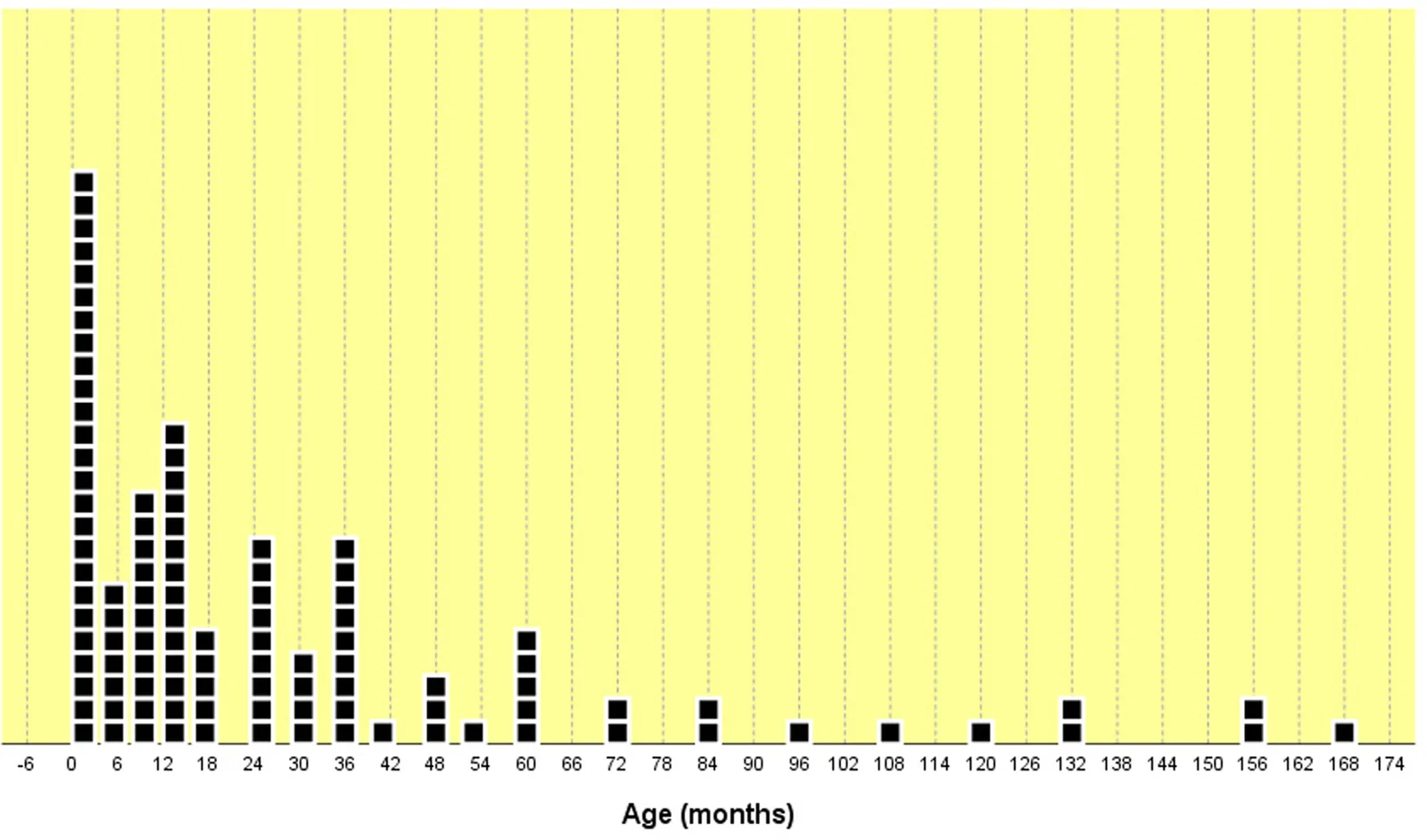
Distribution of patients by age (in months).

A more detailed analysis by months revealed that 15.8% of cases were diagnosed within the first month of life, with a gradual increase in diagnosis frequency observed in the first year, reaching a peak of 17.8% by the 12^th^ month. The mean age in months at diagnosis was 28.4±36.7 months (range: 1 day to 168 months) (Fig. **1**).

### 3.3 Pathologic Findings

Among the 106 patients, 61 cases (57.5%) were confirmed to have Hirschsprung disease with absent ganglion cells, while 45 cases (42.5%) showed normal ganglion cells.

### 3.4 Site of Involvement

For clarity, anatomical classification of affected segments was aligned with standard international categories: short-segment (rectosigmoid), classic-segment (rectum to descending colon), long-segment (proximal to splenic flexure), and total colonic aganglionosis.

The most commonly affected anatomical sites were:

- Colon: 35.8% (n=38)
- Rectum: 23.6% (n=25)
- Sigmoid colon: 12.3% (n=13)
- Recto-sigmoid junction: 10.4% (n=11)
- Bowel: 7.5% (n=8)
- Colostomy sites: 4.7% (n=5)
- The ilium and appendix were affected in 2.8% (n = 3) each.

**Fig. (2):**
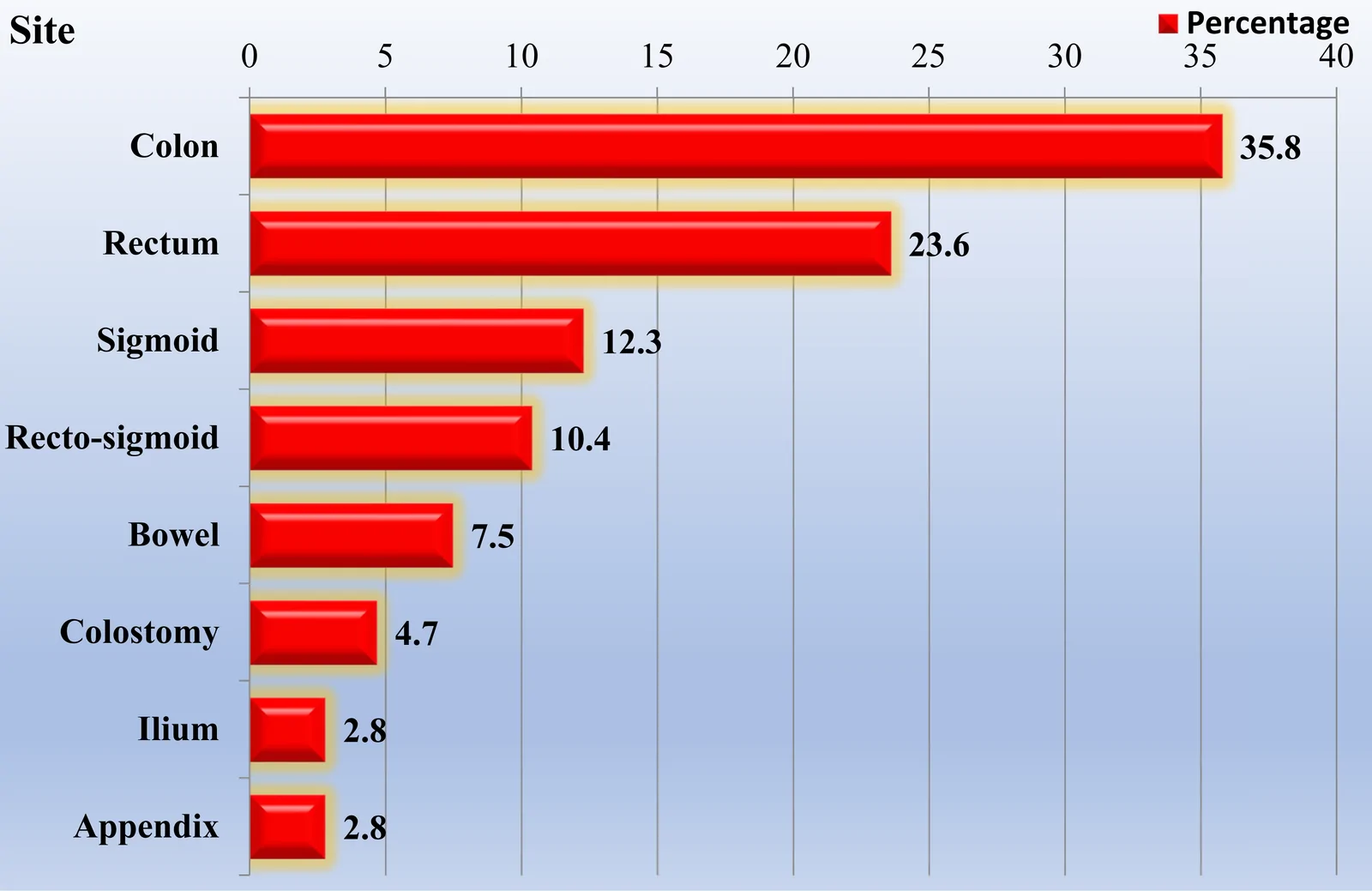
Percentage distribution of affected anatomical sites in patients with suspected Hirschsprung’s Disease.

Statistical analysis indicated a significant difference in site distribution between patients with and without Hirschsprung disease (*P* = 0.010, Cramér’s V = 0.15), which reflects a small but meaningful effect size. The rectum was the most commonly affected site among patients with confirmed Hirschsprung disease (Table **1** and Fig. **2**).

### 3.5 Surgical Interventions

The most common surgical procedures performed included:

- Rectal biopsy: 64.2% (n=68)
- Colon resection: 35.8% (n=38)

**Fig. (3):**
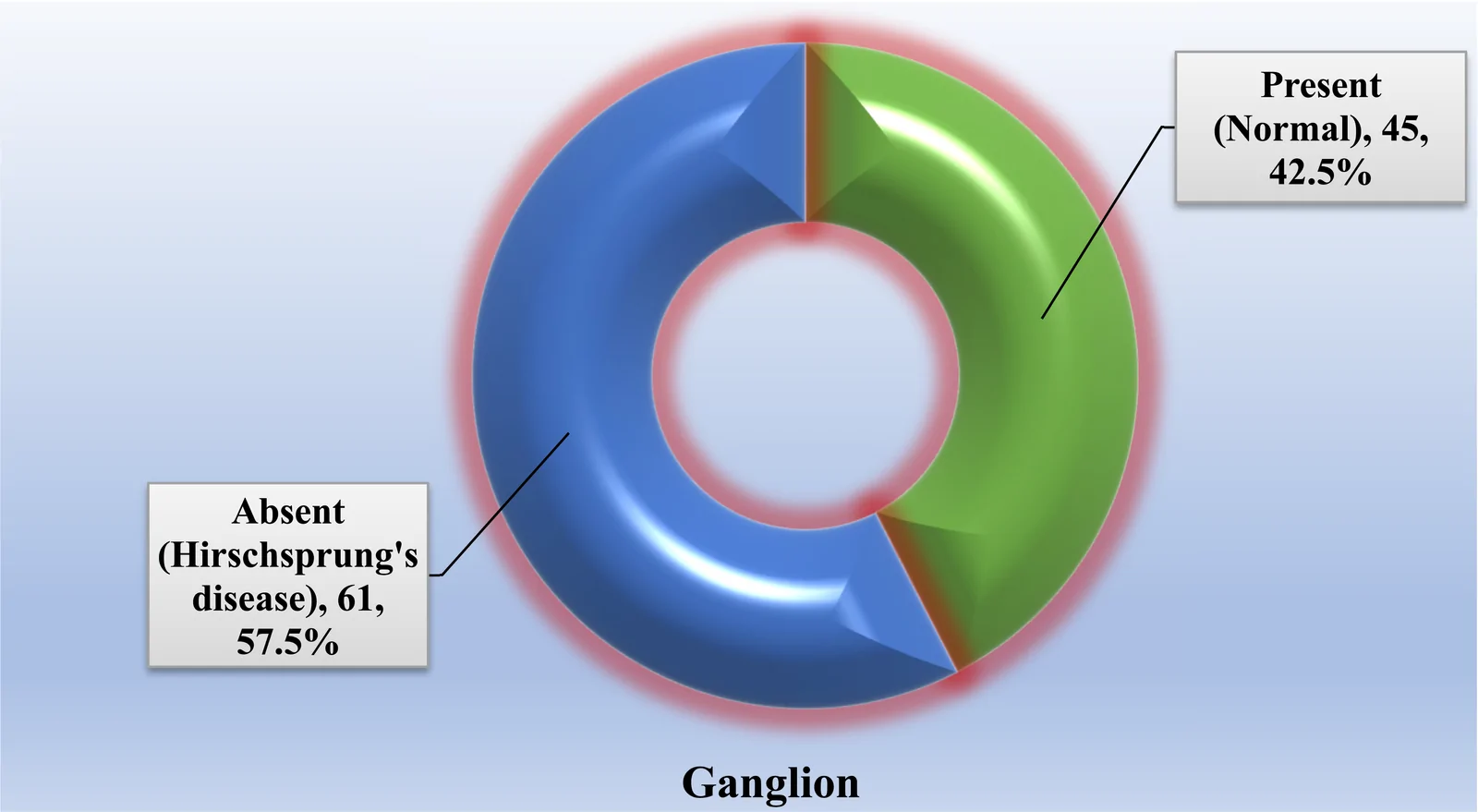
Distribution of ganglion cell presence among patients suspected of hirschsprung’s disease.

A statistically significant difference was found in the type of surgical intervention performed between the two groups (*P* = 0.046, Cramér’s V = 0.19), indicating a small effect size. Rectal biopsy was more common in patients with absent ganglion cells (Table **2** and Fig. **3**).

### 3.6 Association of Age and Gender with Ganglion Status

- **Age:** There was no statistically significant association between age and the presence or absence of ganglion cells (*P*=0.319). Patients with Hirschsprung disease had a slightly lower mean age (2.2±2.7 years) compared to those with normal ganglion cells (2.8±3.4 years), as shown in Fig. (**4**).
- **Gender:** No significant gender differences were observed in ganglion cell distribution (*P* = 0.308), although a higher proportion of males (68.8%) were found to have absent ganglion cells compared to females (31.1%), as shown in Table **3**.

## 4 DISCUSSION

The findings of our study provide a comprehensive overview of the incidence and characteristics of Hirschsprung disease at the Central Pediatrics Teaching Hospital in Iraq over a 10-year period. This timeframe was utilized in several related studies [47-52]. Our results align with previously published studies, particularly in terms of the male predominance observed, which has been consistently reported in the literature with male-to-female ratios ranging from 3:1 to 4:1. This suggests that genetic or hormonal factors may play a role in the higher susceptibility of males to Hirschsprung disease [53, 54].

In this study, 40.6% of Hirschsprung Disease (HD) cases were diagnosed after infancy, highlighting a significant delay in detection. While HD typically presents in the neonatal period, delayed diagnosis is not uncommon in low- and middle-income countries (LMICs) such as Iraq. Several socioeconomic and systemic factors likely contribute to these delays. These include limited access to specialized pediatric surgical care, geographic disparities between urban and rural health services, and financial constraints that hinder early referral and investigation. In many regions of Iraq, particularly in peripheral provinces, there is a shortage of pediatric surgeons and trained pathologists, which may delay both rectal biopsy and definitive diagnosis. Additionally, cultural perceptions and low parental awareness of congenital diseases can result in delayed health-seeking behavior. According to a study by Al-musawi *et al*., delays in accessing tertiary care for congenital disorders in Iraq are significantly associated with parental education level and regional health infrastructure deficiencies [55, 56]. Moreover, ongoing challenges in the healthcare system, exacerbated by years of conflict and underfunding, may further contribute to prolonged diagnostic timelines. Addressing these gaps requires not only improved diagnostic facilities at the district level but also community-based awareness programs and timely referral pathways for neonates with suspected HD [57-61].

The anatomical distribution of affected sites in our cohort, with the colon (35.8%) and rectum (23.6%) being the most commonly involved areas, is consistent with findings from previous studies conducted in both Western and Middle Eastern populations. Studies from Europe and North America have reported similar anatomical patterns, with rectosigmoid involvement being the most frequent. This consistency reinforces the notion that the disease pathology follows a universal pattern, despite geographical variations [62, 63].

Our study identified a significant association between surgical interventions and the presence of Hirschsprung disease, with rectal biopsy being the most commonly performed procedure. This finding is consistent with prior research, which emphasizes the diagnostic accuracy of rectal biopsy as the gold standard for confirming the disease. Additionally, our study highlighted the importance of colon resection in managing severe cases, aligning with surgical practices observed in previous reports [64-66].

Compared to other studies, the percentage of cases with absent ganglion cells (57.5%) is within the range reported in similar hospital-based studies, further validating the reliability of our findings. However, as shown in Fig. (**4**) and Table **3**, variations in ganglion status across different age groups and genders suggest the need for further research to understand the disease's progression and presentation in different populations [67].

## CONCLUSION

Hirschsprung disease continues to be extremely hard to diagnose and treat, especially in places with few resources and slow referral processes. The epidemiological characteristics, pathological discoveries, and clinical interventions related to HD at Iraq's Central Pediatrics Teaching Hospital are highlighted in this ten-year retrospective study. The disease's genetic and embryological consistency across populations is highlighted by the observed male predominance and frequent involvement of the colon and rectum, which is consistent with the international literature.

Histopathological confirmation, particularly through rectal biopsy, is crucial for establishing a conclusive diagnosis, as evidenced by the strong association between the absence of ganglion cells and the type and anatomical location of surgical intervention. These results underscore the importance of incorporating early diagnostic techniques into pediatric practice, particularly in primary care settings, to facilitate prompt referrals.

Furthermore, the significant percentage of cases diagnosed after infancy points to a crucial gap in early detection and highlights the need for increased professional and public awareness of HD symptoms. Results can be improved by strengthening pediatric pathology training, improving intraoperative decision-making using frozen sections, and ensuring that the right histological documentation is done.

Hirschsprung disease continues to be a major contributor to intestinal blockage in children in Iraq, which puts a strain on tertiary care facilities. Therefore, to minimize delays and lower morbidity, it is advised to make strategic investments in diagnostic infrastructure, interdisciplinary collaboration, and capacity building in pediatric surgery and pathology.

## STUDY LIMITATIONS

It is important to recognize the various limitations of this study. First, the results may not be applicable to larger populations or other healthcare settings, as this was a single-center retrospective study.

## GENETIC ASPECTS

Although the pathogenesis of Hirschsprung Disease is strongly associated with mutations in genes such as RET, EDNRB, and SOX10, this study did not include genetic analysis due to limited resources and institutional constraints. Therefore, any reference to genetic mechanisms remains theoretical and based on previous literature. Future studies incorporating molecular and genetic testing are recommended to validate these associations in the local population.

Another limitation relates to immunohistochemical (IHC) analysis. While IHC was performed for most patients, the lengthy study duration and resource constraints resulted in incomplete data for some cases. This may have influenced the confirmation of borderline histological findings. Finally, although the sample size is statistically justified, it may not adequately represent the disease's rare presentations or epidemiological diversity.

## CLINICAL IMPLICATIONS AND RECOMMENDATIONS

Recommendations: Based on our experiences and discussions from the clinics and laboratories, the following are our recommendations for practicing pediatric pathologists working in a pediatrics training center: (1) At least one skilled and trained pediatric pathologist overseeing the specialized pathologies in the departmental laboratories to help in making the appropriate diagnosis; (2) Permanent cooperation between the cliniccians, especially surgeons, and pediatric pathologists; (3) Documentation and descriptions of the macroscopic pathologies before processing the tissues are necessary because color differences are beneficial. By the same token, the existence of thin muscles and mucus depletion is a crucial key. Finally, (4) frozen sections are sometimes required to assess the margins in an intraoperative setting. The completeness of the pre-pull-through barium enema examination should be emphasized and confirmed.

## Figures and Tables

**Fig. (4):**
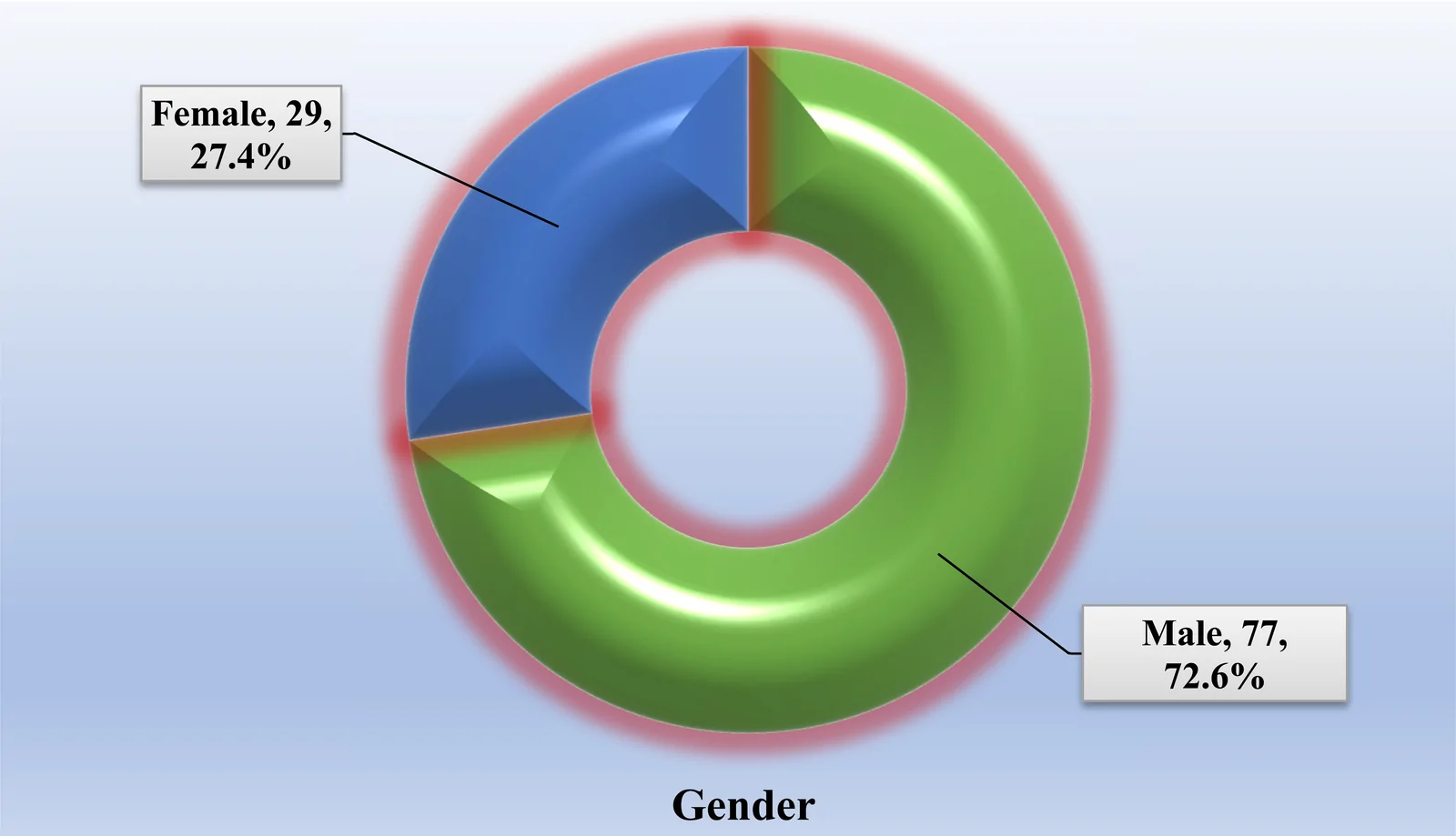
Gender distribution of patients with suspected hirschsprung’s disease.

**Table 1:** Distribution of affected sites, performed operations, and ganglion cell findings in study patients.

-		No.	%
**Site**	Bowel	8	7.5
	Ilium	3	2.8
	Colon	38	35.8
	Appendix	3	2.8
	Sigmoid	13	12.3
	Recto-sigmoid	11	10.4
	Rectum	25	23.6
	Colostomy	5	4.7
**Operation**	Colon resection	38	35.8
	Rectal biopsy	68	64.2
**Ganglion**	Present (Normal)	45	42.5
	Absent (Hirschsprung's disease)	61	57.5

**Table 2:** Association between affected site or operation type and presence or absence of ganglion cells in patients suspected of hirschsprung’s disease.

-		**Ganglion**				-	-
		Present (Normal)		Absent (Hirschsprung's Disease)		*P* Value	Effect Size (Cramér’s V)
		No.	%	No.	%		
**Site**	Bowel	4	50.0	4	50.0	0.010*	0.15 (small)
	Ilium	2	66.7	1	33.3	-	-
	Colon	21	55.3	17	44.7	-	-
	Appendix	1	33.3	2	66.7	-	-
	Sigmoid	8	61.5	5	38.5	-	-
	Recto-sigmoid	4	36.4	7	63.6	-	-
	Rectum	2	8.0	23	92.0	-	-
	Colostomy	3	60.0	2	40.0	-	-
**Operation**	Colon resection	21	55.3	17	44.7	0.046*	0.19 (small)
	Rectal biopsy	24	35.3	44	64.7	-	-

**Note:** *Significant difference between percentages using Pearson Chi-square test (χ^2^-test) at 0.05 level.

**Table 3:** Relationship between ganglion cell presence and patient age or gender in suspected hirschsprung’s disease cases.

-		**Ganglion**				-
		Present (Normal)		Absent (Hirschsprung's Disease)		*P* Value
		No.	%	No.	%	
**Age (years)**	<1year	16	37.2	27	62.8	0.693
	1---	15	46.9	17	53.1	-
	3---	7	36.8	12	63.2	-
	6---	3	60.0	2	40.0	-
	=>9years	4	57.1	3	42.9	-
	Mean±SD (Range)	2.8±3.4 (1D-13Y)		2.2±2.7 (2D-14Y)		0.319
**Gender**	Male	35	45.5	42	68.8%	0.308
	Female	10	34.5	19	31.1%	-

**Note:** *Significant difference between percentages using Pearson Chi-square test (χ^2^-test) at 0.05 level.

## Data Availability

All data generated or analyzed during this study are included in this published article.

## LIST OF ABBREVIATIONS

CD56 (NCAM): Neural Cell Adhesion Molecule (Immunohistochemical Marker)
EDN3: Endothelin 3
EDNRB: Endothelin Receptor Type B
HD: Hirschsprung Disease
IHC: Immunohistochemistry
LMICs: Low- and Middle-Income Countries
RET: RET Proto-Oncogene
SD: Standard Deviation

## STANDARDS OF REPORTING

STROBE guidelines were followed.
