# Supplementary material for: Incidence of Hirschsprung Disease at the Central Pediatrics Teaching Hospital in Iraq: A Pathological Overview
Source: Endocr Metab Immune Disord Drug Targets. 2025 Oct 20;26:E18715303401696. doi: 10.2174/0118715303401696251002054047 (PMC13563429; doi:10.2174/0118715303401696251002054047)
Supplement: Supplementary file 1 [file EMIDDT-26-E18715303401696_SD1.pdf]

Supplementary Material

Incidence of Hirschsprung Disease at the Central Pediatrics Teaching Hospital in Iraq: A Pathological Overview

Ikram Fakhri Abed Al-Zughaibi[1,2], Nada K. Mehdi[3,4] and Hany Akeel Al-Hussaniy[2,*]

[1]Pathologist/Arab Board of Health Specializations in Anatomic Pathology (ABHS-APath), Al Zahraa Teaching Hospital in Kut, Iraq; [2]Department of pharmacology, College of Pharmacy, University of Al-Nisour, 10001, Baghdad, Iraq; [3]Senior pathologist/Central Pediatrics Teaching Hospital, Baghdad, Iraq; 4 Iraqi Medical Research Center, Baghdad, Iraq

This checklist confirms that the study complies with SAGER guidelines.

Question	Response (Yes/No/N.A.)	Comments/Details
1. Was the sex of research subjects reported in the title or abstract?	Yes	Mentioned in abstract and results (male-to-female ratio).
2. Were the methods used to determine sex and gender clearly described?	Yes	Sex recorded as part of demographic data.
3. Were sex and/or gender differences considered in study design and analysis?	Yes	Sex-specific analysis of ganglion absence provided.
4. Were sex- or gender-based analyses reported separately where appropriate?	Yes	Results section contains sex-specific comparisons.
5. Was discussion of sex/gender implications included in interpretation of results?	Yes	Discussed male predominance and possible genetic factors.

STROBE Statement Checklist for Observational Studies.

Item No.	Recommendation	Page No. in Manuscript
1	1. Title and abstract: Indicate study's design in title or abstract.	1 (Title, Abstract)
2	2. Background/rationale: Explain scientific background and rationale.	2-4 (Introduction)
3	3. Objectives: State specific objectives and hypotheses.	2-4 (Introduction)
4	4. Study design: Present key elements early in the paper.	5 (Materials and Methods)
5	5. Setting: Describe setting, locations, and relevant dates.	5 (Materials and Methods)

6	6. Participants: Eligibility criteria, sources, and methods of selection.	5 (Materials and Methods)
7	7. Variables: Clearly define all outcomes, exposures, predictors, etc.	5 (Methods)
8	8. Data sources/measurement: Describe sources and methods of data collection.	5 (Methods)
9	9. Bias: Describe any efforts to address potential sources of bias.	5 (Discussion)
10	10. Study size: Explain how study size was determined.	5 (Methods)
11	11. Quantitative variables: Explain handling in analyses.	5 (Methods)
12	12. Statistical methods: Describe all statistical methods and control for confounding.	6 (Methods)
13	13. Participants: Report numbers at each stage of study.	7-12 (Results)
14	14. Descriptive data: Characteristics of participants and exposures/confounders.	7-12 (Results)
15	15. Outcome data: Report numbers of outcome events.	7-12 (Results)
16	16. Main results: Estimates and confidence intervals.	7-12 (Results)
17	17. Other analyses: Subgroups and sensitivity analyses.	7-12<br>(Results)
18	18. Key results: Summarize key results with reference to objectives.	12,13(Discussion)
19	19. Limitations: Discuss study limitations.	13 (Discussion)
20	20. Interpretation: Give a cautious interpretation considering objectives and limitations.	13,14 (Conclusion)
21	21. Generalizability: Discuss the generalizability of the results.	14 (Conclusion)
22	22. Funding: Give source of funding and role of funders.	15 (Funding)

Patient ID	Initials	Age (months)	Gender	Ganglion Status	Affected Site	Surgical Procedure
1	IH	75	M	Absent	Sigmoid	Rectal biopsy
2	MY	83	M	Absent	Sigmoid	Rectal biopsy
3	QS	34	M	Absent	Rectum	Rectal biopsy
4	JQ	1	M	Absent	Recto-sigmoid	Rectal biopsy
5	CB	50	M	Absent	Colon	Rectal biopsy
6	FE	36	M	Absent	Rectum	Rectal biopsy
7	WL	51	M	Absent	Colon	Rectal biopsy
8	ZB	40	M	Absent	Colon	Rectal biopsy
9	DW	1	M	Absent	Appendix	Rectal biopsy
10	BD	1	M	Absent	Colon	Rectal biopsy
11	WI	51	M	Absent	Rectum	Rectal biopsy
12	JM	1	M	Absent	Colostomy	Rectal biopsy
13	KJ	56	M	Absent	Colon	Rectal biopsy
14	PI	16	M	Absent	Rectum	Rectal biopsy
15	RY	1	M	Absent	Colon	Rectal biopsy
16	UP	1	M	Absent	Colon	Rectal biopsy
17	YT	51	M	Absent	Rectum	Rectal biopsy
18	PY	4	M	Absent	Appendix	Rectal biopsy
19	RG	11	M	Absent	Rectum	Rectal biopsy
20	OJ	73	M	Absent	Rectum	Rectal biopsy
21	IM	1	M	Absent	Recto-sigmoid	Rectal biopsy
22	CD	36	M	Absent	Colon	Rectal biopsy
23	QR	54	M	Absent	Rectum	Rectal biopsy
24	IK	33	M	Absent	Sigmoid	Rectal biopsy
25	ED	91	M	Absent	Rectum	Rectal biopsy
26	YX	72	M	Absent	Rectum	Rectal biopsy
27	ZM	77	M	Absent	Rectum	Rectal biopsy
28	PU	22	M	Absent	Colon	Rectal biopsy
29	IM	1	M	Absent	Colon	Rectal biopsy
30	IY	24	M	Absent	Bowel	Rectal biopsy
31	KG	8	M	Absent	Sigmoid	Rectal biopsy
32	VJ	71	M	Absent	Recto-sigmoid	Rectal biopsy
33	LM	1	M	Absent	Colon	Rectal biopsy
34	VR	5	M	Absent	Ilium	Rectal biopsy
35	LJ	60	M	Absent	Recto-sigmoid	Rectal biopsy
36	NH	51	M	Absent	Bowel	Rectal biopsy
37	UV	1	M	Absent	Recto-sigmoid	Rectal biopsy
38	IJ	22	M	Absent	Colon	Rectal biopsy
39	KW	13	M	Absent	Colon	Rectal biopsy
40	GP	1	M	Absent	Rectum	Rectal biopsy
41	HK	62	M	Absent	Sigmoid	Rectal biopsy
42	FZ	97	M	Absent	Colon	Rectal biopsy

43	EI	75	M	Absent	Colon	Rectal biopsy
44	EX	66	M	Absent	Colon	Rectal biopsy
45	IZ	1	M	Absent	Sigmoid	Rectal biopsy
46	ML	84	M	Absent	Rectum	Rectal biopsy
47	NT	39	M	Absent	Colostomy	Rectal biopsy
48	DJ	1	M	Absent	Recto-sigmoid	Rectal biopsy
49	DV	73	M	Absent	Bowel	Rectal biopsy
50	RP	4	M	Absent	Colon	Rectal biopsy
51	ZU	1	M	Absent	Colon	Rectal biopsy
52	VA	55	M	Absent	Ilium	Rectal biopsy
53	HC	1	M	Absent	Rectum	Rectal biopsy
54	EC	11	M	Absent	Rectum	Rectal biopsy
55	LI	61	M	Absent	Colostomy	Rectal biopsy
56	DN	4	M	Absent	Rectum	Rectal biopsy
57	XY	15	M	Absent	Recto-sigmoid	Rectal biopsy
58	RZ	72	M	Absent	Rectum	Rectal biopsy
59	EK	13	M	Absent	Colon	Rectal biopsy
60	BX	115	M	Absent	Bowel	Rectal biopsy
61	IH	40	M	Absent	Colon	Rectal biopsy
62	FC	8	M	Present	Colon	Colon resection
63	VW	46	M	Present	Sigmoid	Rectal biopsy
64	TC	20	M	Present	Rectum	Colon resection
65	AV	1	M	Present	Colon	Rectal biopsy
66	WH	78	M	Present	Colon	Colon resection
67	QT	13	M	Present	Colon	Rectal biopsy
68	VA	1	M	Present	Recto-sigmoid	Rectal biopsy
69	LQ	13	M	Present	Colon	Rectal biopsy
70	PQ	62	M	Present	Colon	Colon resection
71	KJ	11	M	Present	Colon	Rectal biopsy
72	MK	1	M	Present	Appendix	Colon resection
73	HI	1	M	Present	Colon	Rectal biopsy
74	KS	29	M	Present	Rectum	Rectal biopsy
75	HX	8	M	Present	Colon	Colon resection
76	FK	35	M	Present	Colon	Colon resection
77	KW	69	M	Present	Colon	Rectal biopsy
78	JC	1	F	Present	Colon	Rectal biopsy
79	PH	1	F	Present	Colon	Rectal biopsy
80	UO	48	F	Present	Colon	Colon resection
81	GT	23	F	Present	Rectum	Rectal biopsy
82	CI	1	F	Present	Rectum	Rectal biopsy
83	PF	12	F	Present	Colon	Rectal biopsy
84	GE	1	F	Present	Bowel	Colon resection
85	KN	17	F	Present	Colon	Rectal biopsy
86	XG	67	F	Present	Colon	Rectal biopsy

87	UI	5	F	Present	Rectum	Colon resection
88	MS	2	F	Present	Ilium	Colon resection
89	GW	25	F	Present	Recto-sigmoid	Rectal biopsy
90	JA	1	F	Present	Bowel	Colon resection
91	IU	6	F	Present	Rectum	Rectal biopsy
92	OA	1	F	Present	Appendix	Colon resection
93	CQ	44	F	Present	Colon	Rectal biopsy
94	PT	1	F	Present	Colostomy	Colon resection
95	HD	42	F	Present	Rectum	Rectal biopsy
96	ZX	1	F	Present	Colon	Colon resection
97	MF	57	F	Present	Colostomy	Colon resection
98	DG	1	F	Present	Recto-sigmoid	Colon resection
99	FX	18	F	Present	Colon	Colon resection
100	UX	68	F	Present	Recto-sigmoid	Colon resection
101	XU	25	F	Present	Appendix	Rectal biopsy
102	VJ	38	F	Present	Colon	Colon resection
103	FG	59	F	Present	Colon	Rectal biopsy
104	HP	1	F	Present	Colon	Rectal biopsy
105	VD	12	F	Present	Bowel	Colon resection
106	TE	1	F	Present	Colon	Colon resection
